# Frailty index is useful for predicting postoperative morbidity in older patients undergoing gastrointestinal surgery: a prospective cohort study

**DOI:** 10.1186/s12893-022-01471-9

**Published:** 2022-02-16

**Authors:** Chaoyang Gu, Anqing Lu, Chen Lei, Qingbin Wu, Xubing Zhang, Mingtian Wei, Ziqiang Wang

**Affiliations:** Department of Gastrointestinal Surgery, West China Hospital, Sichuan University, No. 37 Guo Xue Alley, Chengdu, 610041 China

**Keywords:** Older patients, Frailty index, Gastrointestinal surgery, Postoperative morbidity

## Abstract

**Background:**

Many assessment tools have been used to identify frail surgical patients. This study was designed to explore the prediction value of the frailty index (FI) for postoperative morbidity in older patients undergoing elective gastrointestinal surgery.

**Methods:**

Between January 2019 and September 2020, we conducted a prospective study in our hospital, and patients aged over 65 years were enrolled. The FI assessment was conducted by two specialist nurses based on the 38-item scale, and patients were considered frail if the FI score was ≥ 0.25. The primary outcome was 30-day postoperative morbidity. Univariable and multivariable analyses were used to find the risk factors related to postoperative morbidity.

**Results:**

A total of 246 consecutive patients were enrolled, for whom the median age was 72.0 [interquartile range (IQR): 67.0–77.0] years old, and 175 (71.1%) were male. Of these, 47 (19.1%) were frail. Patients with frailty were associated with older age (*p* < 0.001), higher American Society of Anesthesiologists (ASA) grade (*p* = 0.006), lower body mass index (*p* = 0.001), lower albumin (*p* = 0.003) and haemoglobin (*p* < 0.001) levels, increased blood loss (*p* = 0.034), increased risk of postoperative morbidity (*p* < 0.001), increased median length of stay (*p* = 0.017), and increased median postoperative hospital stay (*p* = 0.003). Multivariable analysis revealed that ASA grade [odds ratio (OR): 2.59, 95% confidence interval (CI) 1.19–5.64, *p* = 0.016], FI score (OR 7.68, 95% CI 3.19–18.48, *p* < 0.001) and surgical complexity (OR 22.83, 95% CI 5.46–95.51, *p* < 0.001) were independent predictors of 30-day postoperative morbidity. However, for patients with major surgery, FI score was the only independent predictor (OR 8.67, 95% CI 3.23–23.25, *p* < 0.001).

**Conclusion:**

Frailty was associated with adverse perioperative outcomes, and the 38-item FI scale was a useful frailty screening tool for older patients undergoing elective gastrointestinal surgery. For patients with major surgery, frailty was a more reliable predictor of postoperative 30-day morbidity than age and ASA grade.

**Supplementary Information:**

The online version contains supplementary material available at 10.1186/s12893-022-01471-9.

## Introduction

Surgical treatment for older patients is increasingly prevalent as the older population is growing at an unprecedented rate. According to a systematic review of 70 studies, the prevalence of frailty in the older people undergoing general surgery ranged from 8% to 77.8% [1]. Frailty is commonly defined as a state of reduced physiologic capacity and increased susceptibility to disability caused by age-related loss of physical, cognitive, social, and psychological functions [2, 3]. The older patients living with frailty have limited physiological reserve, hence, are susceptible to surgical stress. Therefore, 25% to 50% postoperative adverse outcomes in older people were resulted from the concomitant frailty [4]. In addition, the presence of frailty before surgery was a strong and objective predictor of postoperative morbidity, mortality, discharge disposition, and health service resource utilisation for a variety of surgeries including gastrointestinal surgeries [1, 5–13]. Consequently, it is increasingly important to assess the functional status and screen for frailty for older patients before surgery, because evidence for preoperative optimization showed prehabilitation and other modalities could improve the patients’ reserve to cope with the stress of surgery [14].

There are two accepted paradigms of frailty: phenotypic construct, and deficit accumulation model. The phenotype construct is based on a cluster of signs and symptoms such as self-reported exhaustion, slowed performance (by walking speed), weakness (by grip strength), unintentional weight loss (4.5 kg in the past year), and low physical activity. The deficit accumulation model, contrarily, is quantified based on the number rather than the nature of health problems, along with biochemical and physiological impairments.⁠ An overlap exists between the two constructs, their sum contributing to a risk state [14]. The gold standard to define frailty in patients is based on the comprehensive geriatric assessment (CGA), which includes activities of daily living (ADL), physical, psychosocial, comorbidity, cognition state, and functional tests. However, CGA is time-consuming and needs professional geriatricians. Therefore, many other instruments had been developed to assess frailty, the Fried Phenotype and its modifications were most prevalent, followed by the clinical frailty scale (CFS), and a physical measure of frailty (gait speed, timed get up and go, handgrip strength, short physical performance battery). However, Fried Phenotype and physical measure of frailty were more concentrated on physical conditions, and CFS was too subjective. Nevertheless, frailty index (FI) scale basing on the accumulation of deficits, was an objective and clinically practical tool that was also commonly used [1]. FI scale also had many versions, such as Groningen Frailty Indicator (15 items) [15], G-8 (8 items) [16], and the latest and shortest version consisted of only 5 comorbidities (mFI-5) [17].

In 2008, Searle et al. developed a standard procedure for FI assessment with 40 items, and it had been simplified to 38 items by Munster et al.[18, 19]. However, this scale had never been used in elective gastrointestinal surgery. Therefore, we decided to conduct a prospective study to explore the value of this FI scale in predicting postoperative morbidity for older patients undergoing gastrointestinal surgery.

## Materials and methods

### Study population

From January 2019 to September 2020, patients aged over 65 years and undergoing elective gastrointestinal surgery in the Department of Gastrointestinal Surgery, West China Hospital, Sichuan University, were prospectively enrolled. This study was approved by the ethics committee of our hospital [Approval number: 2019 (160)] and had registered in *Clinicaltrails.gov* (NCT03930082). Written informed consent was obtained from each participant, and this work was reported in line with the STROCSS criteria [20].

### Parameters measurement and frailty assessment

Scale proposed by Searle SD et al. and modified by Munster et al. was used for FI assessment, which included 38 items consisting of ADL, comorbidity, physical, psychological, social, and cognitive items (Additional file 1: Table S1) [18, 19]. The frailty assessment was completed within 6 h after admission. Firstly, the principal investigator interviewed every admitted patient, and relevant information, including comorbidity and mini-mental state examination (MMSE) scores were collected. Secondly, a specialist geriatric-trained nurse performed a series of function tests and completed the questionnaire (Additional file 1: Table S1) [18, 19]. Maximal grip strength was measured in the dominant hand using an electronic hand dynamometer. Walking speed (usual and rapid pace) was measured as the fastest time of two measurements. Five non-recordable grip strength and two missing walk time because of inability to walk were scored as positive items for frailty assessment. In addition, we also performed a set of function tests, however, similar to Munster et al., shoulder strength and peak flow measurement were excluded [19]. The FI was calculated by the proportion of positive items to all the 38 items. For the purposes of this study, we defined an FI score < 0.25 as non-frail and a score of ≥ 0.25 as frail [18].

### Data collection and outcomes

C reactive protein (CRP), interleukin 6 (IL-6), albumin, and haemoglobin (Hb) were tested and recorded within one week before surgery. Age, sex, body mass index (BMI), American Society of Anesthesiologists (ASA) grade were also recorded. In addition, we also collected operation time, blood loss, postoperative complications, postoperative intensive care unit (ICU) admission rate, 30-day readmission, 30-day reoperation, length of hospital stay (LOS) and postoperative hospital stay (PHS). Postoperative complications were further defined using the Claviene-Dindo classification system. According to the surgical complexity, hernioplasty and laparoscopic exploratory biopsy were classified as minor surgery, while gastrectomy, colectomy, anterior resection (AR) for cancers in the stomach and colorectum were classified as major surgery.

The primary outcome was 30-day postoperative morbidity. Thus, we further explored the predicting value of the FI score in different complexity of surgeries.

### Statistical analysis

Continuous variables were reported as median [interquartile range (IQR) 25–75%], while categorical variables were shown as frequency and proportions. Chi-square (χ^2^) test, independent t-test, and Wilcoxon rank-sum test were used to compare demographics and outcomes between different groups. *p*-value < 0.05 was considered statistically significant. Correlation analysis was performed to explore the association between FI score and age. Univariable and multivariable logistic regression analyses were used to estimate the impact of frailty on postoperative morbidity, and odds ratio (OR) was calculated. To explore the impact of FI score on 30-day postoperative morbidity, Univariable and multivariable analyses were performed. To unify variable types for multivariable analyses, continuous variables such as age, blood loss, and operative time were divided into dichotomous variables by appropriate cutoff values. Finally, eight factors that could affect postoperative complications, including age, gender, FI score, ASA grade, surgical approach, surgical complexity, blood loss, and operative time were included in the analysis. Alpha was set at 0.05 and 95% confidence intervals (CIs) were reported. All statistical analyses were performed using SPSS, version 24 (IBM Corp., Armonk, NY).

## Results

### Baseline characteristics and clinical outcomes

**Fig. 1:**
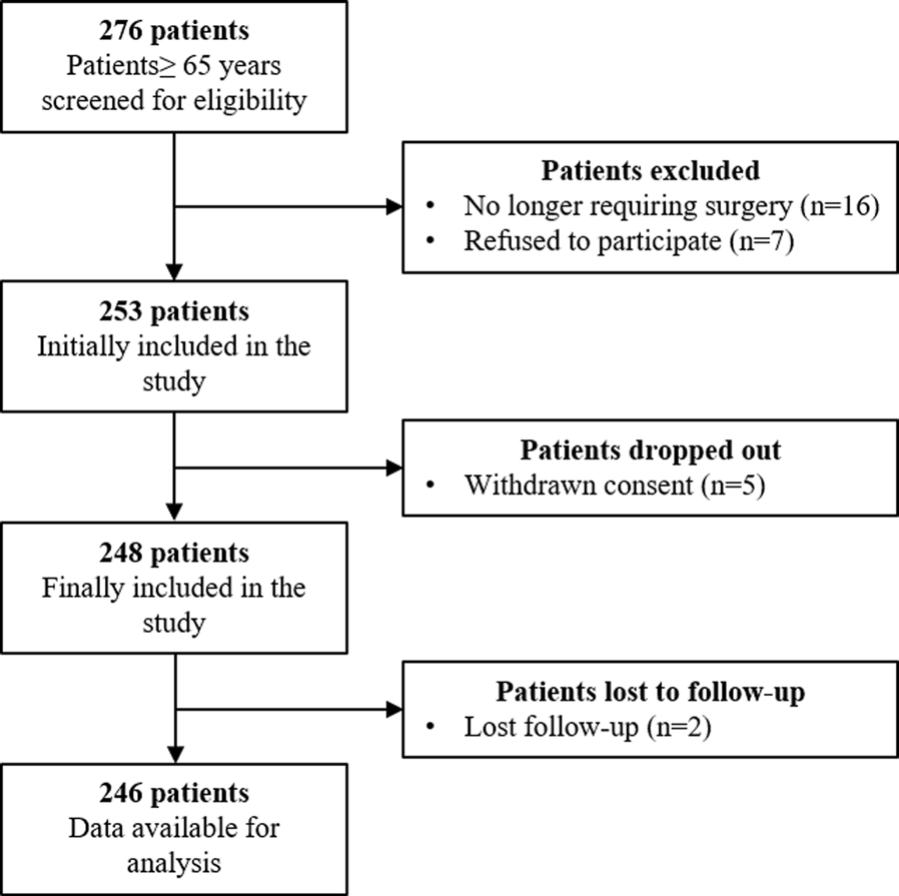
Flow chart of patients enrolled

**Fig. 2:**
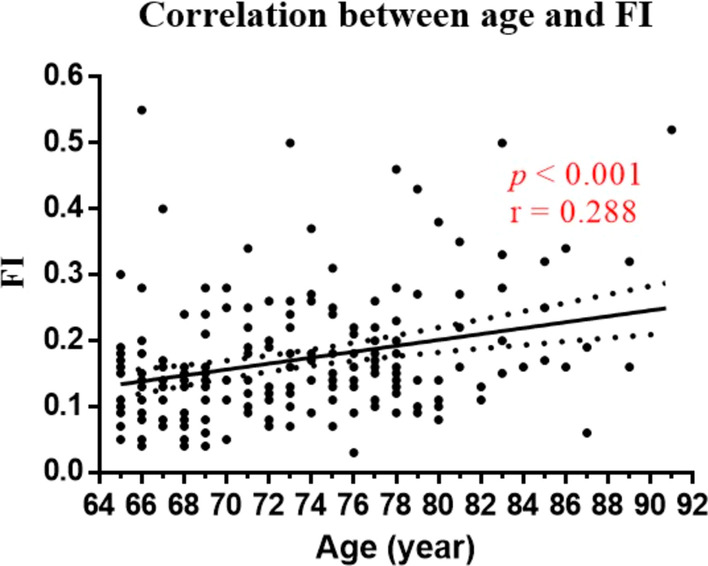
Correlation analysis between age and frailty index

A total of 276 patients were screened for eligibility, and 246 consecutive patients aged over 65 years were included in the analysis (Fig. 1). The median age was 72 (IQR 67.0–77.0) years old, and 175 (71.1%) patients were male. Based on the FI score of ≥ 0.25, 47 patients (19.1%) were frail. Correlation analysis showed that increasing correlated with the FI score (*p* < 0.001, r = 0.288, Fig. 2). Besides, frail patients had lower median BMI (23.4 vs 22.1 kg/m^2^, *p* = 0.005), lower median albumin level (41.1 40.2 g/L, *p* = 0.010), lower median Hb level (132 vs 118 g/L, *p* < 0.001), and higher ASA grade (II vs III, *p* = 0.006) (Table 1).

**Table 1:** Patient baseline characteristics with or without frailty

	No. of patients (n = 246)	Non-frail (n = 199)	Frail (n = 47)	*p*
Age (years)^a^	72 (67.0–77.0)	71.0 (67.0–76.0)	74.0 (70.0–81.0)	0.001
Sex (n, %)				0.876
F	71 (28.9)	57 (28.6)	14 (29.8)	
M	175 (71.1)	142 (71.4)	33 (70.2)	
BMI (kg/m^2^)^a^	23.4 (20.8–25.1)	23.4 (21.2–25.3)	22.1 (19.9–24.3)	0.005
Albumin (g/L)^a^	40.9 (38.7–43.3)	41.1 (39.3–43.4)	40.2 (37.6–42.2)	0.010
Hb (g/L)^a^	130.5 (115.0–140.3)	132.0 (118.0–143.0)	118.0 (96.0–113.0)	< 0.001
CRP (g/L)^a^	3.6 (2.3–7.3)	3.8 (2.4–6.7)	3.3 (2.1–26.8)	0.712
IL-6 (ng/L)^a^	3.9 (3.1–12.2)	4.0 (3.1–12.4)	3.8 (2.4–10.7)	0.945
ASA grade (n, %)				0.006
II	158 (64.2)	136 (68.3)	22 (46.8)	
III	88 (35.8)	63 (31.7)	25 (53.2)	

*BMI* body mass index, *Hb* hemoglobin, *CRP* C-reactive protein, *IL-6* interleukin-6, *ASA* American Society of Anesthesiologists

^a^Data showed as median (interquartile range 25–75%)

One hundred and six patients (43.1%) underwent minor surgeries, and 140 patients (56.9%) underwent major surgery (Table 2). Of the 106 patients with minor surgery, 95 (89.6%) patients underwent hernioplasty, 11 (10.4%) patients underwent laparoscopic exploratory biopsy (Additional file 1: Table S2). Among the 140 patients undergoing major surgery, 22 (15.7%) underwent total gastrectomy, 9 (6.4%) underwent distal gastrectomy, 4 (2.9%) underwent proximal gastrectomy, 37 (26.4%) underwent colectomy, and 68 (48.6%) underwent anterior resection (AR) (Table 3).

**Table 2:** Intra-and post-operative outcomes of all patients

	No. of patients (n = 246)	Non-frail (n = 199)	Frail (n = 47)	*p*
Surgical approach (n, %)				0.700
Open + converted	183 (74.4)	147 (73.9)	36 (76.6)	
Laparoscopy	63 (25.6)	52 (26.1)	22 (23.4)	
Surgical complexity (n, %)				0.085
Minor^a^	106 (43.1)	91 (45.7)	15 (31.9)	
Major^b^	140 (56.9)	108 (54.3)	32 (68.1)	
Operative time (min)^c^	147.5 (44.0–210.0)	137 (40.0–200.0)	170 (60.0–240.0)	0.105
Blood Loss (ml)^c^	30.0 (5.0–70.0)	30.0 (5.0–50.0)	50.0 (10.0–100.0)	0.034
ICU admission (n, %)	6 (2.4)	3 (1.5)	3 (6.4)	0.086^d^
Morbidity (n, %)	59 (24.0)	32 (16.1)	27 (57.4)	< 0.001
Mortality (n, %)	2 (0.8)	1 (0.5)	1 (2.1)	0.346^d^
Readmission (n, %)	5 (2.0)	3 (1.5)	2 (4.3)	0.244^d^
Reoperation (n, %)	5 (2.0)	3 (1.5)	2 (4.3)	0.244^d^
LOS (day)^c^	8.0 (4.0–11.0)	8.0 (3.0–10.0)	10.0 (7.0–13.0)	0.017
PHS (day)^c^	6.0 (1.0–7.3)	6.0 (1.0–7.0)	7.0 (5.0–9.0)	0.003

^a^Hernioplasty and laparoscopic exploration biopsy

^b^Gastrectomy, colectomy, anterior resection (AR) for cancers in the stomach and colorectum

^c^Data showed as median (interquartile range 25–75%)

^d^Fisher exact test

**Table 3:** Intra-and post-operative outcomes of patients with major surgery

	No. of patients (n = 140)	Non-frail (n = 108)	Frail (n = 32)	*p*
Surgical approach (n, %)				0.150
Open + Converted	90 (64.3)	66 (61.1)	24 (75.0)	
Laparoscopy	50 (35.7)	42 (38.9)	8 (25.0)	
Procedure				0.020
Gastrectomy	35 (25.0)	22 (20.4)	13 (40.6)	
Colectomy + AR	105 (75.0)	86 (79.6)	19 (59.4)	
Operative Time (min)^a^	191.0 (158.0–247.3)	180.0 (153.0–247.3)	209.0 (166.0–252.5)	0.159
Blood Loss (ml)^a^	50.0 (40.0–100.0)	50.0 (30.0–100.0)	100.0 (50.0–100.0)	0.018
ICU admission (n, %)	6 (4.3)	3 (2.8)	3 (9.4)	0.132*
Morbidity (n, %)	55 (39.3)	30 (27.8)	25 (78.1)	< 0.001
Mortality (n, %)	2 (1.4)	1 (0.9)	1 (3.1)	0.406*
Readmission (n, %)	5 (3.6)	3 (2.8)	2 (6.3)	0.321*
Reoperation (n, %)	5 (3.6)	3 (2.8)	2 (6.3)	0.321*
LOS (day)^a^	10.0 (8.3–13.0)	10.0 (8.0–12.0)	11.0 (10.0–16.0)	0.032
PHS (day)^a^	7.0 (6.0–9.0)	7.0 (6.0–8.0)	9.0 (7.0–12.8)	0.001

*ICU* intensive care unit, *LOS* length of hospital stay, *PHS* length of postoperative hospital stay, *AR* anterior resection

^a^Data showed as median (interquartile range 25–75%)

*Fisher exact test

Six patients (2.4%) were admitted to ICU postoperatively, four patients were transferred to ICU for respiratory support due to difficulty in removing endotracheal intubation after operation, and two patients were transferred to ICU due to circulatory instability caused by anastomotic leakage and postoperative bleeding. Five patients (2.0%) were readmitted following repeat surgery for complications. Of the five patients, three were due to anastomotic leakage, one was due to postoperative bleeding, and the other was due to incision dehiscence. A total of 81 complications occurred in 59 patients within 30 days postoperatively, most of those (55/59, 93.2%) were patients in major surgery group (Table 4). Postoperative complications were classified by the Clavien–Dindo scoring system, and did not demonstrate any differences in grade III and above complications between frail and non-frail patients (6.4% [3/47] vs. 3.0% [6/199], *p* = 0.629) (Table 5).

**Table 4:** Details of 30-day postoperative complications

	No. of patients (n = 246)	Non-frail (n = 199)	Frail (n = 47)
Postoperative pneumonia (n, %)	29 (11.7)	14 (7.0)	15 (31.9)
SSI (n, %)	13 (5.3)	8 (4.0)	5 (10.6)
POI (n, %)	9 (3.7)	4 (2.0)	5 (10.6)
Anastomotic leakage (n, %)	6 (2.4)	3 (1.5)	3 (6.4)
PRI (n, %)	5 (2.0)	3 (1.5)	2 (4.3)
Postoperative bleeding (n, %)	5 (2.0)	2 (1.0)	3 (6.4)
Urinary retention (n, %)	3 (1.2)	2 (1.0)	1 (2.1)
DVT (n, %)	2 (0.8)	1 (0.5)	1 (2.1)
Cardiac-cerebral vascular events (n, %)	2 (0.8)	0 (0)	2 (4.3)
Other (n, %)	7 (2.8)	5 (2.5)	2 (4.3)
Total (n, %)*	59 (23.9)	32 (16.1)	27 (57.4)

*SSI* surgical site infection, *POI* postoperative ileus, *PRI* progressive renal insufficiency; *DVT* deep venous thrombosis

*A total of 81 complications occurred in 59 patients

**Table 5:** Claviene-Dindo classification of postoperative complications

	No. of patients (n = 246)	Non-frail (n = 199)	Frail (n = 47)	*p*
I	19 (7.7)	9 (4.5)	10 (21.3)	0.629
II	31 (12.6)	17 (8.5)	14 (29.8)	
≥ III	9 (3.7)	6 (3.0)	3 (6.4)	

### Association of frailty with overall intra-and post-operative outcomes and who underwent major and minor surgery

When compared to non-frail patients, patients with frailty were significantly associated with more median blood loss (30.0 ml vs 50.0 ml, *p* = 0.034), higher postoperative morbidity (16.1% vs 57.4%, *p* < 0.001), longer median LOS (8.0 days vs 10.0 days, *p* = 0.017) and PHS (6.0 days vs 7.0 days, *p* = 0.003) (Table 2).

Subgroup analysis showed that following major surgery, patients with frailty had higher median blood loss (50.0 ml vs 100.0 ml, *p* = 0.017), higher postoperative morbidity (27.8% vs 78.1%, *p* < 0.001), longer LOS (10.0 days vs 11.0 days, *p* = 0.032) and PHS (7.0 days vs 9.0 days, *p* = 0.001). These were similar to overall analysis (Table 3). However, frailty was not associated with intra-and postoperative parameters for patients following minor surgery (Additional file 1: Table S2).

### Independent predictors analysis of 30-day postoperative morbidity

Binary Univariable logistic regression analysis showed FI score (OR 7.05, 95% CI 3.53–14.06, *p* < 0.001), ASA grade (OR 3.05, 95% CI 1.67–5.57, *p* < 0.001), surgical complexity (OR 16.50, 95% CI 5.75–47.39, *p* < 0.001), operative time (OR 4.11, 95% CI 2.22–7.58, *p* < 0.001), and blood loss (OR 5.29, 95% CI 2.76–10.12, *p* < 0.001) were associated with postoperative morbidity. However, multivariable analysis identified the FI score, ASA grade, as well as surgical complexity, having the strongest association with postoperative morbidity (ORs, 7.677, 2.592 and 22.830, respectively; 95% CIs, 3.19–18.48, 1.19–5.64 and 5.46–95.51; *p* < 0.001, *p* = 0.016 and *p* < 0.001) (Table 6).

**Table 6:** Univariable and multivariable logistic regression analysis of all patients for morbidity

Variables	Univariable (n = 246)			Multivariable (n = 246)		
	OR	95% CI	*p* value	OR	95% CI	*p* value
Gender (male vs female)	0.60	0.32–1.11	0.101	0.79	0.36–1.72	0.548
Age (≥ 75 vs < 75)	1.17	0.64–2.14	0.607	1.573	0.67–3.69	0.298
FI score (Frail vs Non-frail)	7.05	3.53–14.06	< 0.001	7.677	3.19–18.48	< 0.001
ASA grade (III vs II)	3.05	1.67–5.57	< 0.001	2.592	1.19–5.64	0.016
Surgical approach (laparoscopic vs open)	1.24	0.65–2.39	0.518	0.905	0.37–2.23	0.827
Surgical complexity (major vs minor)	16.50	5.75–47.39	< 0.001	22.830	5.46–95.51	< 0.001
Operative time (≥ 180 min vs < 180 min)	4.11	2.22–7.58	< 0.001	0.997	0.42–2.38	0.994
Blood loss (≥ 50 ml vs < 50 ml)	5.29	2.76–10.12	< 0.001	1.121	0.41–3.09	0.825

The same analysis was also performed for patients with major surgery. Univariable analysis identified age (OR 2.66, 95% CI 1.23–5.75, *p* = 0.013), FI score (OR 9.29, 95% CI 3.64–23.72, *p* < 0.001), and ASA grade (OR 3.05, 95% CI 1.50–6.21, *p* = 0.002) were associated with postoperative morbidity, while multivariable analysis found FI score was the only independent predictor (OR 8.669, 95% CI 3.233–23.245, *p* < 0.001) (Table 7).

**Table 7:** Univariable and multivariable logistic regression analysis of patients with major operations for morbidity

Variables	Univariable (n = 140)			Multivariable (n = 140)		
	OR	95% CI	*p* value	OR	95% CI	*p* value
Gender (male vs female)	0.88	0.44–1.78	0.729	0.86	0.38–1.99	0.730
Age (≥ 75 vs < 75)	2.66	1.23–5.75	0.013	2.03	0.82–5.05	0.129
FI score (Frail VS Non-frail)	9.29	3.64–23.72	< 0.001	8.67	3.23–23.25	< 0.001
ASA grade (III VS II)	3.05	1.50–6.21	0.002	2.27	0.98–5.25	0.055
Surgical approach (laparoscopic vs open)	0.71	0.34–1.45	0.341	0.93	0.37–2.34	0.817
Operative time (≥ 180 min vs < 180 min)	1.56	0.78–3.12	0.213	1.11	0.45–2.72	0.825
Blood loss (≥ 50 ml vs < 50 ml)	1.59	0.74–3.43	0.239	0.94	0.32–2.72	0.907

*OR* odds ratio, *CI* confidence interval, *FI* frailty index, *ASA* American Society of Anesthesiologists

## Discussion

To our best knowledge, this was the first study which prospectively validated the effectiveness of FI (38 items) in older patients undergoing elective gastrointestinal surgery. In this study, we found frailty was associated with older age, lower BMI, lower ALB and Hb level, and higher ASA grade. In addition, patients with frailty also had more intra-operative blood loss, higher incidence of postoperative complications, longer LOS and PHS. Moreover, the result of multivariable analysis for postoperative morbidity indicated that frailty assessed by 38-item FI scale was a more reliable predictor than age and ASA grade.

As expected, FI score increased steadily with age, as showed in Fig. 1, however, the small r value (0.288) did not indicate a strong correlation between age and FI score as frailty was a state of pathological aging, and aging alone was not equal to frailty [21]. Frailty was also associated with a low-grade chronic pro-inflammatory state characterised by increased levels of CRP and IL-6, and could further result in anaemia [22–24]. Although there was no difference in CRP and IL-6 levels between the frail and non-frail groups in this study, frailty was significantly associated with a lower Hb level (*p* < 0.001).

Previous studies demonstrated patients with frailty were associated with adverse postoperative outcomes, including a higher incidence of morbidity, mortality, and ICU admission across surgical specialties [10, 13, 25–30]. In our study, although there was no significant difference between frail and non-frail patients regardless of overall analysis or subgroup analysis for major surgery, the ICU admission rate and mortality of frail patients were higher than non-frail patients. Moreover, the incidence of 30-day postoperative complications in patients with frailty was significantly higher than that in non-frail patients (57.1% vs 16.1%, p < 0.001). In addition, for patients with major surgery, multivariable analysis identified that only FI independently predicted postoperative morbidity, suggesting that frailty assessed by 38-item FI was a more reliable predictor than age and ASA grade, which was supported by Miller et al. [31].

A meta-analysis for patients with general surgery showed frail patients had a longer LOS than non-frail patients (9.6 vs 6.4 days, 95% CI 6.2–12.9) [32]. In a prospective study in patients who followed a standardised enhanced recovery pathway, Keller et al. demonstrated a strong association between longer LOS and frailty [33]. Our study demonstrated similar findings of longer median LOS and PHS in frail patients.

There was no doubt that recovery after major surgery will need more physiological reserve and it was challenging for older patients. The 38-item FI score could therefore help in categorising older patients requiring major surgery into different risk groups in terms of 30-day postoperative morbidity, LOS, and PHS. Although the 38-item FI was not associated with any perioperative adverse events in patients requiring minor surgery, the incidence of adverse events in patients with minor surgery was fairly low (3.8%). We believe that more subjects are probably needed to verify the effectiveness of 38-item FI in this group of patients in the future. In addition, these results also indicate that more sensitive assessment tools are needed for patients with minor gastrointestinal surgery because high sensitivity ensures frail patients can be correctly screened out [34].

Previous studies showed that preoperative prevention could reduce postoperative complications, preoperative exercise, management of comorbidity and nutrition could improve the postoperative outcomes of older patients [35–37]. Therefore, the 38-item FI scale could be used preoperatively to modify practice and potentially improve outcomes. It could be a useful tool for screening patients with frailty who need preoperative prevention, given that FI score was associated with adverse postoperative outcomes and was reliable for predicting complications. In addition, it was also helpful for preoperative informed consent and better allocated of postoperative support. In our institution, the FI score is now calculated in all elective cases. For those frail patients, the multidisciplinary care team can preemptively arrange postoperative nursing care, physical therapy, social work, and discharge disposition. Thus, a different clinical pathway may be more appropriate for frail patients. We believe there is potential to improve postoperative outcomes. Future prospective studies are needed to evaluate the implementation and outcomes of these altered pathways.

There are several limitations in the present study. Patients enrolled in the cohort mixed major and minor gastrointestinal surgeries, which had different incidences of postoperative adverse events. Nevertheless, subgroup analysis for patients with major surgery also revealed a clear correlation between FI score and postoperative adverse outcomes. Furthermore, the present study is a single-centre study with a small sample size, which also limits the validation efficiency for 38-item FI scale. In addition, the 38-item FI scale treat cancer as a positive item which would inevitably lead to higher FI score in patients with cancers. Finally, frailty assessment by this scale is more time-consuming, so it is not suitable for emergency and outpatient patients. However, for inpatients preparing for surgery, it is worthwhile to use this scale for a comprehensive assessment. Further studies are needed to verify its superiority before its generalization.

## Conclusion

Frailty was associated with adverse postoperative outcomes, and FI scale (38 items) is a useful tool for screening out frail patients from older patients undergoing elective gastrointestinal surgery. Our study also demonstrate that frailty has a robust impact on postoperative morbidity. Moreover, for patients with major gastrointestinal surgery, the FI score is a more reliable predictor of postoperative morbidity than age and ASA grade.

## Supplementary Information

**Additional file 1: Table S1.** Frailty index scale (38 items). **Table S2.** Intra-and post-operative outcomes of patients with minor surgery.

## Data Availability

The data used and analyzed during the current study are available from the corresponding author on reasonable request.

## Abbreviations

CGA: Comprehensive geriatric assessment
ADL: Activities of daily living
CFS: Clinical Frailty Scale
FI: Frailty index
MMSE: Mini-mental state examination
CRP: C reactive protein
IL-6: Interleukin 6
Hb: Haemoglobin
BMI: Body mass index
ASA: American Society of Anesthesiologists
ICU: Intensive care unit
LOS: Length of hospital stay
PHS: Length of postoperative hospital stay
AR: Anterior resection
IQR: Interquartile range
OR: Odds ratio
HR: Hazard ratio
CI: Confidence interval
