# Supplementary material for: Frailty index is useful for predicting postoperative morbidity in older patients undergoing gastrointestinal surgery: a prospective cohort study
Source: BMC Surg. 2022 Feb 16;22:57. doi: 10.1186/s12893-022-01471-9 (PMC8851713; doi:10.1186/s12893-022-01471-9)
Supplement: Supplementary file 1 — Additional file 1: Table S1. Frailty index scale (38 items). Table S2. Intra-and post-operative outcomes of patients with minor surgery. [file 12893_2022_1471_MOESM1_ESM.docx]

**Supplementary table 1:** Frailty index scale (38 items)

| **Frailty Instrument** | **Components** | | **Measurement** | | | | **Classification** |
| --- | --- | --- | --- | --- | --- | --- | --- |
| **Frailty Index** | 38 items, each scored 0 - 1 | | | | | | Total score of positive items / Total available items = index score  Index score of ≥0.25: frail. |
|  | ADL/IADL | Help Bathing  Help Dressing  Help getting in/out of Chair  Help Walking around house  Help Eating  Help Grooming  Help Using Toilet  Help up/down Stairs  Help lifting 10 lbs  Help Shopping  Help with Housework  Help with meal Preparations  Help taking Medication  Help with Finances | | | Yes = 1, No = 0 | |  |
|  | Physical | - Lost more than 10 lbs in last year - Stayed in Bed at least half the day due to health (in last month) - Cut down on Usual Activity (in last month) | | | Yes = 1, No = 0 | |  |
|  |  | - Walk outside | | | <3 days = 1, ≤ 3 days = 0 | |  |
|  | Psychosocial | - Self Rating of Health | | | Poor = 1  Fair = 0.75  Good = 0.5  Very Good = 0.25  Excellent = 0 | |  |
|  |  | - How Health has changed in last year | | | Worse = 1  Better/Same = 0 | |  |
|  |  | - Feel Everything is an Effort - Feel Depressed - Feel Happy - Feel Lonely - Have Trouble getting going | | | Most of time = 1  Sometime = 0.5  Rarely = 0 | |  |
|  | Comorbidity | High blood pressure  Heart attack  Congestive Heart Failure  Stroke  Cancer  Diabetes  Arthritis  Chronic Lung Disease | | | Yes = 1  Suspect = 0.5  No = 0 | |  |
|  | Function test | MMSE | | <10 = 1  11–17 = 0.75  18–20 = 0.5  20–24 = 0.25  >24 =0 | | |  |
|  |  | Grip Strength (GS in kg) | | Men  GS ≤ cutoff =1  BMI ≤ 24, GS ≤ 29  BMI 24.1–28, GS ≤ 30  BMI >28, GS ≤ 32 | | Women  GS ≤ cutoff =1  BM≤ 23 GS ≤ 17  BMI 23.1–26, GS ≤ 17.3  BMI 26.1–29, GS ≤ 18  BMI>29, GS ≤ 21 |  |
|  |  | Body Mass Index (BMI) | | <18.5, ≥ 30 = 1  25-<30 = 0.5 | | |  |
|  |  | Rapid pace Walk, 20 feet | | >10 seconds =1 | | |  |
|  |  | Usual pace Walk, 20 feet | | >16 seconds =1 | | |  |

This scale is cited from Munster et al. (Clin Kidney J. 2016;9(4):606-10.)

Supplementary table 2 Intra-and post-operative outcomes of patients with minor surgery

|  | No. of patients (n= 106) | Non-frail (n= 91) | Frail (n= 15) | *p* |
| --- | --- | --- | --- | --- |
| Surgical approach (n, %) |  |  |  | 0.324 |
| Open + Converted | 93 (87.7) | 81 (89.0) | 12 (80.0) |  |
| Laparoscopy | 13 (12.3) | 10 (11.0) | 3 (20.0) |  |
| Procedure |  |  |  |  |
| Open/ Laparoscopic hernioplasty | 95 (89.6) | 83 (91.2) | 12 (80.0) | 0.187 |
| Laparoscopic exploratory biopsy | 11 (10.4) | 8 (8.8) | 3 (20.0) |  |
| Operative Time (min)^#^ | 39.5 (30.0-60.0) | 38.0 (30.0-60.0) | 44.0 (32.0-60.0) | 0.577 |
| Blood Loss (ml)^#^ | 5.0 (5.0-10.0) | 5.0 (5.0-10.0) | 5.0 (5.0-10.0) | 0.495 |
| ICU admission (n, %) | 0 (0.0) | 0 (0.0) | 0 (0.0) | **-** |
| Morbidity (n, %) | 4 (3.8) | 2 (2.2) | 2 (13.3) | 0.095^※^6 |
| Mortality (n, %) | 0 (0.0) | 0 (0.0) | 0 (0.0) | - |
| Readmission (n, %) | 0 (0.0) | 0 (0.0) | 0 (0.0) | - |
| Reoperation (n, %) | 0 (0.0) | 0 (0.0) | 0 (0.0) | - |
| LOS (day)^#^ | 3.0 (3.0-7.0) | 3.0 (3.0-7.0) | 6.0 (3.0-7.0) | 0.339 |
| PHS (day)^#^ | 1.0 (1.0-4.0) | 1.0 (1.0-4.0) | 2.0 (1.0-5.0) | 0.331 |

ICU intensive care unit, LOS length of hospital stay, PHS length of postoperative hospital stay. ^#^ Data showed as median (interquartile range:25%-75%). ^#^ Data showed as median (interquartile range:25%-75%). ^※^ Fisher exact test.
